# Neoadjuvant Treatment in Patients with HER2-Positive Breast Cancer

**DOI:** 10.1155/2013/362467

**Published:** 2013-05-23

**Authors:** Katarina Sevcikova, Bibiana Vertakova-Krakovska, Stanislav Spanik

**Affiliations:** ^1^1st Department of Oncology, Faculty of Medicine, Comenius University, Spitalska 24, 813 72 Bratislava, Slovakia; ^2^Department of Clinical Oncology, St. Elizabeth Cancer Institute, Heydukova 10, 812 50 Bratislava, Slovakia

## Abstract

Approximately 20%–25% of patients with breast cancer demonstrate amplification of the human epidermal receptor type 2 (HER2) gene, resulting in an overexpression of the HER2 receptor. This overexpression is associated with aggressive disease, relatively poor prognosis, and worse clinical outcomes. Neoadjuvant therapy is the standard treatment in patients with locally advanced, inflammatory, or inoperable primary breast cancer. It is generally used to downstage the tumors and therefore to improve surgical options including breast-conserving surgery rather than mastectomy. It has been confirmed that patients with pathological complete response (pCR) to neoadjuvant treatment have better disease-free survival (DFS) and overall survival (OS). Neoadjuvant treatment can also serve as in vivo test of sensitivity to the used therapeutic regimen. The preferred neoadjuvant approach to patients with HER2-positive breast cancer is a sequential anthracycline-taxane-based chemotherapy in combination with trastuzumab. Addition of other anti-HER2 agents has increased pCR rate up to 75% and will probably become a new therapeutic direction. In the first part of this paper, we summarize the information about HER2-positive breast cancer, the various treatment possibilities, and the results of the major neoadjuvant trials. The second part focuses on the data concerning the importance of pCR and the potential risk of cardiotoxicity associated with this treatment.

## 1. Introduction

HER2 belongs to the epidermal growth factor receptor (EGFR/ErbB) family of receptor tyrosine kinases. This family consists of four receptors—HER1, HER2, HER3, and HER4—which are involved in regulating cell growth, survival, and differentiation. HER receptors are inactive monomers, and to activate signaling pathways, they have to undergo dimerization. Pairing among the molecules of the same HER receptor is called homodimerization; pairing of different HER receptor subtypes is called heterodimerization. HER dimerization leads to activation of two important signaling pathways—PI3K/Akt and Ras/Raf/MEK/MAPK [1]. HER2 is always in active conformation and it is a preffered partner for other HER receptors, especially HER3 and the HER2-HER3 dimer is an important oncogenic unit that signals constitutively to PI3K and Akt [2]. 

All breast cancers should be evaluated for HER2 overexpression. HER2 testing can be done by targeting protein and gene. The most widely used methods to detect HER2 amplification are immunohistochemistry (IHC) and fluorescence in-situ hybridization (FISH). Amplification or overexpression of HER2 is present in around 22% of early breast cancers, 35% of locally advanced and metastatic tumors, and 40% of inflammatory breast cancers, and is associated with aggressive disease and poor prognosis [3, 4]. In normal cells, only few HER2 molecules are present at the cell surface, so growth signals are relatively weak and under control. When HER2 is overexpressed, multiple HER2 heterodimers are formed. Therefore, cell signaling is much stronger, leading to increased responsiveness to growth factors and malignant growth [5]. This explains why HER2 overexpression is an indicator of poor prognosis in breast tumors.

Neoadjuvant therapy is the standard modality in patients with locally advanced, inflammatory, and inoperable primary breast cancer [6–9]. It can significantly reduce the tumor size and make patients with breast cancer suitable for surgical resection or in some cases for breast-conserving surgery rather than mastectomy. Many studies have confirmed that patients with pCR to neoadjuvant treatment have better long-term outcomes [10–15]. Today, the neoadjuvant treatment is considered at least as effective as the adjuvant treatment in terms of survival in patients with locally advanced breast cancer [16]. The achieved response to the neoadjuvant treatment offers also valuable information about the sensitivity to the used therapeutic regimen, which is important for further management of the patient.

## 2. Treatment Modalities in Neoadjuvant Setting

### 2.1. Chemotherapy

The most commonly chemotherapy regimens used in the neoadjuvant setting contain an anthracycline (adriamycin or epirubicin) in combination or sequentially administered with taxanes (paclitaxel or docetaxel). Anthracyclines can be combined with cyclophosphamide and fluoropyrimidine. Many combinations have been tested but no specific regimen is considered to be clearly superior.

### 2.2. Biological Treatment

#### 2.2.1. Trastuzumab

Trastuzumab is a humanized recombinant monoclonal antibody specifically directed against HER2 receptor, and, according to the international panel on neoadjuvant therapy, it should be part of the neoadjuvant treatment regimen in patients with HER2-positive breast cancer [17]. Trastuzumab is the first developed agent targeting HER2 pathway and its binding to the extracellular domain of HER2 receptor leads to inhibition of tumor cell growth. Mechanisms of its antitumor action include the following: antibody-dependent cell-mediated cytotoxicity, inhibition of cleavage of the extracellular domain of the HER2 receptor, inhibition of ligand-independent HER2 receptor dimerization, inhibition of downstream signaling pathways and angiogenesis, induction of cell-cycle arrest and apoptosis, and interference with DNA repair [18–20].

#### 2.2.2. Lapatinib

Lapatinib is an orally active, small molecule which reversibly inhibits HER1 and HER2 tyrosine kinase. This inhibition leads to blockage of MAPK and PI3/Akt signaling pathways, resulting in growth arrest and/or apoptosis, as observed in cell line and xenografts models [21]. Some data indicate that lapatinib can also block HER2-HER3 mediated cell growth [22, 23]. Lapatinib as a small molecule can penetrate the blood-brain barrier and, therefore, is being studied for therapy and prevention of brain metastases.

#### 2.2.3. Pertuzumab

Pertuzumab is the humanized monoclonal antibody that binds to dimerization domain II of HER2 receptor, which is necessary for HER2 activation and cell signaling. Clinically, the most important action of pertuzumab is inhibition of HER2-HER3 dimerization. Pertuzumab affects important signaling pathways that mediate cell proliferation and synergistically with trastuzumab inhibits breast tumor cells survival [24–29].

### 2.3. Other Agents under Evaluation in HER2-Positive Breast Cancer

Many other agents are being assessed in clinical trials, for example, trastuzumab-DM1 (conjugate of monoclonal antibody trastuzumab, the cytotoxic agent maytansinoid, and a highly stable thioeter linker—so-called—“targeted chemotherapy”), other tyrosine kinase inhibitors, heat shock protein 90 inhibitors, mTOR inhibitors, inhibitors of angiogenesis, and vaccines.

## 3. Which Treatment Regimen Is the Most Appropriate in HER2-Positive Breast Cancer in Neoadjuvant Setting?

We currently do not know the exact answer to this question. A number of chemotherapy + trastuzumab combinations have proven good response rates with good tolerability. However, several aspects have yet to be clearly defined: which regimen has superior efficacy; how long the neoadjuvant therapy should last; if anthracyclines should be incorporated or not; and if they should only be used sequentially or also concurrently with trastuzumab.

Sequential anthracycline-taxane-based chemotherapy in combination with trastuzumab gives a pCR of 40% compared with a pCR of 17% with the chemotherapy alone [30]. The new potential means of treatment presents chemotherapy in combination with dual HER2 blockage based on trastuzumab plus lapatinib/pertuzumab, with encouraging results as will be mentioned below.

## 4. Trastuzumab in Neoadjuvant Setting: Summary of the Major Trials

The use of chemotherapy in combination with trastuzumab in a neoadjuvant setting has been investigated in a number of studies.

In the first reported randomized trial, Buzdar et al. from the MD Anderson Cancer Center, evaluated patients with HER2-positive, early-stage operable breast cancer, who were assigned to receive four cycles of fluorouracil + epirubicin + cyclophosphamide with or without trastuzumab weekly [31, 32]. The addition of trastuzumab to neoadjuvant chemotherapy significantly increased the pCR rate—65.2%—in patients treated with trastuzumab versus 26.3% in the chemotherapy arm alone. These results led to a premature closure of the study and such high pCR rates have not been observed in other trials. There were no cases of cardiac clinical dysfunctions or cardiac deaths in the group treated with anthracycline and trastuzumab concurrently.

Additional data on trastuzumab in neoadjuvant setting in patients with newly diagnosed locally advanced breast cancer come from the Neoadjuvant Herceptin (*NOAH*) trial [33]. This trial included 228 HER2-positive patients and assessed efficacy and safety of sequential doxorubicin + paclitaxel followed by paclitaxel, then cyclophosphamide + methotrexate + 5-fluorouracil, with or without trastuzumab. A third arm included 99 HER2-negative patients, who received the same chemotherapy but without trastuzumab. HER2-positive patients treated with trastuzumab had significantly improved overall response rate (ORR) (87% versus 74%; *P* = 0.009) and pCR rate (43% versus 22%; *P* = 0.0007) compared to patients treated with chemotherapy alone. There was observed also significant improvement in event-free survival in HER2-positive patients who received chemotherapy plus trastuzumab compared with those who received chemotherapy alone (71% versus 56%, HR 0.59; *P* = 0.013).

Another important randomized phase III study is the German Breast Group/Gynecologic Oncology Study Group (*GeparQuattro*) trial, which included 1509 patients with either locally advanced (T3 or T4), hormone receptor-negative or hormone receptor-positive but lymph-node-positive tumors [34, 35]. This trial assessed the incorporation of capecitabine in an anthracycline/taxane-based regimen and the concurrent use of trastuzumab in HER2-positive patients. All patients received four cycles of epirubicin (90 mg/m^2^) and cyclophosphamide (600 mg/m^2^), and they were then assigned to one of three arms: docetaxel (100 mg/m^2^), docetaxel (75 mg/m^2^) + capecitabine (1800 mg/m^2^), or four cycles of docetaxel (75 mg/m^2^) followed by capecitabine (1800 mg/m^2^). Four-hundred forty-five patients were HER2 positive and reached higher pCR compared to the HER2-negative group (31.7% versus 15.7%). In the HER2-positive group, a pCR was observed in 48 (32.9%) of 146 patients in the first arm, 45 (31.3%) of 144 patients in the second arm, and 47 (34.6%) of 136 patients in the third arm. Even in the case of irresponsiveness to epirubicin/cyclophosphamide therapy, the pCR rate was higher in the HER2-positive group. No cardiac failure was observed.

The efficacy and safety of epirubicin and cyclophosphamide followed by paclitaxel and trastuzumab in a neoadjuvant setting in patients with HER2-positive breast cancer were evaluated in the Taxol Epirubicin Cyclophosphamide Herceptin NeOadjuvant study (*TECHNO*) [36]. This multicenter, prospective, open-label, phase II clinical trial enrolled 217 patients who received neoadjuvant epirubicin + cyclophosphamide for four cycles every three weeks followed by paclitaxel (175 mg/m^2^) once every three weeks plus trastuzumab 6 mg/kg every three weeks after a loading dose of 8 mg/kg. Chemotherapy was followed by surgery and trastuzumab was then continued until the completion of 12 months of treatment. The primary endpoint was pCR defined as no residual invasive tumor in breast and lymphatic tissue. A 39 percentage of patients achieved a pCR. Three-year DFS was 88% in patients with pCR compared to 73% in patients without pCR (*P* = 0.01). Similarly, three-year OS was also better (96%) in patients with pCR compared with a rate of 86% in patients without pCR (*P* = 0.025). pCR was the only significant prognostic factor for DFS (hazard ratio (HR) 2.5; 95% CI, 1.2 to 5.1; *P* = 0.013) and OS (HR 4.9; 95% CI, 1.4 to 17.4; *P* = 0.012) in multivariable analysis. Cardiac toxicity was reported in eight patients (3.7%), six of whom presented with an asymptomatic left ventricular ejection fraction decrease and two with symptomatic heart failure.

## 5. Other Anti-HER2 Agents and Dual Anti-HER2 Blockage in Neoadjuvant Setting: Summary of the Major Trials

Lapatinib, a dual tyrosine kinase inhibitor, has been evaluated in a neoadjuvant setting as a single agent or in combination with trastuzumab. The NeoAdjuvant Lapatinib and/or Trastuzumab Treatment Optimisation (*NeoALTTO*) trial included 455 HER2-positive patients who had tumors at least 2 cm in diameter [37]. The patients were randomly assigned to receive lapatinib (1500 mg), trastuzumab (loading dose of 4 mg/kg; subsequent doses of 2 mg/kg), or lapatinib (1000 mg) plus trastuzumab. Anti-HER2 therapy alone was given for 6 weeks and then in combination with weekly paclitaxel (80 mg/m^2^) for 12 weeks, followed by surgery at week 18. After surgery, patients received adjuvant chemotherapy (3 cycles of fluorouracil + epirubicin + cyclophosphamide) followed by the same anti-HER2 treatment as in neoadjuvant setting for 52 weeks. Combination of lapatinib and trastuzumab led to a significantly higher pCR rate (51.3%) than that of the monotherapy arms. The response to lapatinib was numerically lower than to trastuzumab, although the difference did not reach statistical significance. No significant cardiac toxicity was reported. The dual combination was associated with higher toxicity, especially diarrhoea and hepatotoxicity, and more patients discontinued therapy because of adverse events. As confirmed from previous studies, a higher pCR rate was observed in estrogen-receptor- (ER-) negative tumors compared with ER-positive ones.

The comparison of lapatinib versus trastuzumab was the aim of the *GeparQuinto* trial [38]. This randomized phase III study included 620 patients with operable or locally advanced HER2-positive breast cancer. Patients were assigned to receive four cycles of epirubicin (90 mg/m^2^) in combination with cyclophosphamide (600 mg/m^2^) and four cycles of docetaxel (100 mg/m^2^) every 3 weeks, with either trastuzumab (loading dose of 8 mg/kg; subsequent doses of 6 mg/kg) or lapatinib (1000–1250 mg) throughout all cycles before surgery. The results have confirmed a higher pCR rate for the trastuzumab arm (30.3%) compared with that of lapatinib (22.7%). The most common adverse effects associated with lapatinib were diarrhoea and skin toxicity; trastuzumab caused more often oedema and dyspnoea.

Another study comparing these two anti-HER2 agents and their combination is randomized phase II study *CHER-LOB* (Chemotherapy Plus Lapatinib, Trastuzumab or Both in HER2 Positive Breast Cancer) [39]. Patients were assigned to receive weekly trastuzumab, lapatinib (1500 mg), or their combination (lapatinib 1000 mg) concurrently with chemotherapy (weekly paclitaxel dose of 80 mg/m^2^ for 12 weeks followed by fluorouracil + epirubicin + cyclophosphamide for four courses). The results have confirmed higher pCR rate (46.7%) when a combination of both anti-HER2 agents was used. No cardiac failure was observed.

Another anti-HER2 agent, pertuzumab, was studied in the randomized phase III Neoadjuvant Study of Pertuzumab and Herceptin in an Early Regimen Evaluation (*NeoSphere*) [40]. This study included patients with operable, locally advanced, and inflammatory breast cancer, who were assigned to receive docetaxel + trastuzumab, docetaxel + trastuzumab + pertuzumab, docetaxel + pertuzumab, or two monoclonal antibodies, without chemotherapy. The doses were as follows: pertuzumab—840 mg loading dose and 420 mg maintenance; trastuzumab—8 mg/kg loading dose and 6 mg/kg maintenance; and docetaxel—75 mg/m^2^ increasing to 100 mg/m^2^ if the starting dose was well tolerated. After surgery, all patients received 3 cycles of chemotherapy (fluorouracil + epirubicin + cyclophosphamide) and trastuzumab every 3 weeks for one year. The pCR rate was 45.8% and thus significantly higher for combination of docetaxel with both anti-HER2 agents, compared with 29% for the trastuzumab arm (*P* = 0.014) and 24% for the pertuzumab arm (*P* = 0.003). This study also included a chemotherapy-free arm, and pCR in women who received the two anti-HER2 agents without chemotherapy was 16.8%. The tolerability of therapy based on the dual anti-HER2 blockage was high; only one patient developed congestive heart failure with the combination of trastuzumab and pertuzumab.

Dual anti-HER2 blockade in neoadjuvant setting, especially its safety in combination with chemotherapy, was evaluated in the Phase II Trastuzumab plus Pertuzumab in Neoadjuvant HER2-Positive Breast Cancer trial (*TRYPHAENA*) [41]. The study included 225 patients with HER2-positive tumors of at least 2 cm in size, and those patients were randomized to three arms. Arms A and B received three cycles of FEC (fluorouracil, epirubicin, cyclophosphamide) followed by three cycles of docetaxel (75 mg/m^2^ with escalation to 100 mg/m^2^ if tolerated), and they were then randomized to start the combination of pertuzumab and trastuzumab with cycle 1 of FEC (arm A) or with cycle 1 of docetaxel (arm B). Arm C received six cycles of concurrent docetaxel (100 mg/m^2^), pertuzumab plus trastuzumab, and carboplatin (6 AUC). The primary endpoint of cardiac safety was met, with a low incidence of symptomatic and asymptomatic left ventricular systolic dysfunction across all arms. pCR rates were similar across the three arms and regardless of the chemotherapy chosen reached from 57% to 66%. Better results were seen in patients with hormone-negative disease.

## 6. Pathologic Complete Response and Other Markers in HER2-Positive Breast Cancer

The aim of neoadjuvant therapy is the eradication of the disease in breast and regional lymph nodes. The results of recent neoadjuvant studies have shown that patients with pCR have improved DFS and OS [36] and reduced relapse rate [33], and pCR is considered a surrogate marker for outcome in HER2-positive patients treated with chemotherapy and trastuzumab. However, different pCR rates and their impact on long-term prognosis have been observed in patients with hormone-negative and hormone-positive tumors.

The MDACC group has detected [42] that ER-negative disease is associated with higher pCR rates, regardless of the drug regimen or the duration of chemotherapy.

The integrated meta-analysis [43] on data from the German Breast Group and the AGO Breast Group has shown that ER-negative patients have a greater chance of pCR than ER-positive patients [OR 3.2 (95% CI: 2.7–3.8); *P* < 0.0001]. Other important parameters identified as predictors for pCR were HER2-positive disease, higher grade, younger age, non-lobular-type tumors, and smaller tumor size.

More recent pooled analysis of German neoadjuvant studies has also revealed different prognostic value of pCR [44]. In patients with HER2-positive/hormone-negative tumors, pCR was associated with significantly higher DFS compared to no pCR, but, in the group of patients with HER2-positive/hormone-positive tumors, no difference was observed.

It has been shown that the estrogen receptor pathway might be a relevant escape mechanism in “triple-positive” tumors [45]. Accordingly, we have to be careful in using pCR as a marker in the case of triple-positive tumors.

In the case of HER2-positive/ER-negative tumors, the gene expression analysis of 114 samples from the NOAH study revealed that the high expression of the plasma cell metagene and the 8q22 amplicon, and low expression of the insulin-like growth factor metagene, were associated with higher pCR rates in this group of patients treated with trastuzumab and chemotherapy [46].

HER2 overexpression induces an activation of signaling pathways including the PI3K/Akt. Some studies have shown that patients with a low expression of PTEN or PI3K mutations have poorer response and worse clinical outcome with trastuzumab-containing therapy [47–49]. The opposite effect was observed with lapatinib. Therefore, low PTEN expression could be a potential biomarker to select patients resistant to trastuzumab but sensitive to lapatinib.

The extracellular domain of the HER2 protein can be detected in peripheral blood as serum HER2, which can be measured by ELISA. *GeparQuattro* and *GeparQuinto* trials showed a positive association between pCR rates and serum HER2 levels [50, 51]. But the biological relevance of this marker is still unknown.

Other biomarkers such as circulating miR-210 levels [52], fragment C *γ* receptor polymorphisms [53], and genes involved with CD40 signaling [54] have been studied to predict the response to HER2 inhibitors in neoadjuvant setting. But apart from the HER2 positivity, none has been validated yet.

Intensive studies have also been done on the markers potentially associated with resistance to trastuzumab. One is p95HER2 (truncated form of HER2 receptor), which was thought to be associated with resistance to trastuzumab but responsiveness to lapatinib, but the results from the studies are controversial. In the CHER-LOB study, the pCR rate was not different for patients with p95HER2-positive or -negative tumors [55]. On the other hand, in the GeparQuattro study, p95HER2-positive tumors paradoxically showed a significantly higher pCR rate compared with that of p95HER2-negative tumors (59% versus 24%) [56].

Other potential markers of resistance to trastuzumab as p-4EBP1—an activator of the mTOR pathways and ALDH1—a stem cell marker, were investigated in patients included in the GeparQuattro study, but their further validation is needed [57].

## 7. Cardiotoxicity

Cardiac toxicity is an important side effect of trastuzumab treatment and has been observed in patients who received trastuzumab as a single agent or in combination with chemotherapy for metastatic disease and in primary breast cancer [58, 59].

The data concerning cardiac events differ from one clinical study to the next. This is a result of different variables such as applied therapy regimens, inclusion and exclusion criteria, and time interval between anthracycline-based chemotherapy and trastuzumab treatment.

In the two randomized neoadjuvant trials [31, 33], trastuzumab was administered concomitantly with anthracyclines, and although a decrease in left ventricular ejection fraction (LVEF) was observed in 27% of patients, symptomatic cardiac events occurred in only 2% of patients given trastuzumab concurrently with doxorubicin.

It has been proved that administration of trastuzumab concurrent with anthracyclines is associated with an increased risk of cardiac toxicity, but this risk can be manageable if the cumulative dose of anthracycline is kept low and/or less-cardiotoxic anthracyclines are used [33, 60–62].

It is well known that the cardiotoxity of anthracyclines and trastuzumab differs in many ways. Trastuzumab can cause cardiac dysfunction identified as type II, which seems not to be dose related; it is higher when trastuzumab is given concurrently with anthracyclines, it seems to be reversible when trastuzumab is discontinued, and it is mostly medically manageable with regular medication for heart failure.

On the other hand, anthracyclines produce a cardiotoxicity identified as type I, which is dose dependent, not reversible, and results in ultrastructural abnormalities, as observed in myocardial biopsies.

It is very important to consider the risk of cardiac toxicity and reduce it by close monitoring of patients. We should avoid using trastuzumab in patients with a baseline LVEF lower than 50%. Extreme caution should be taken with patients older than 65 and in patients with baseline LVEF 50%–55%. 

We do not know exactly whether typical cardiac risk factors (e.g., hypertension, diabetes, etc.) are also risk factors for trastuzumab-related cardiotoxicity. Seidman et al. [63] performed a multivariate analysis for potential risk factors (e.g., age, hypertension, previous radiation therapy to the chest wall, cumulative anthracycline dose, and baseline LVEF), and age alone (when trastuzumab was administered concomitantly with doxorubicin) was significantly positively associated with the risk of trastuzumab-related cardiotoxicity. All trials with trastuzumab have excluded patients with pre-existing chronic heart failure, and trastuzumab should not be used even in those patients with borderline postchemotherapy LVEF <50%.

Results of retrospective clinical experience studies suggest that advanced age, hypertension, radiation therapy to the left chest wall, or previous exposure to anthracyclines did not result in a higher risk for trastuzumab-related cardiotoxicity [64]. On the other hand, a strong association between baseline LVEF and the risk for cardiac events was confirmed. However, the number of cardiac events was small. Diabetes, history of coronary artery disease, and valvular disease were associated with a higher incidence of cardiac events, although statistical significance was not reached.

These results have to be applied with caution as, in another study, smoking, family history, hypercholesterolemia and diabetes, and radiation to the left side of the chest were not identified as risk factors [65]. Conditions like history of myocardial infarction or angina, uncontrolled hypertension, valvular disease, or arrhythmia exclude patients from the large trials and there is lack of information about potential cardiotoxic effects of trastuzumab in these patients.

For identification of trastuzumab-related cardiotoxicity, all patients treated with this agent should, a part from a clinical examination with electrocardiogram, undergo the measurement of LVEF at baseline of trastuzumab treatment. Furthermore, it is recommended that LVEF be monitored every 3 months during trastuzumab treatment.

Biomarkers such as NT-proBNP and troponin I are being intensively studied as potential parameters for trastuzumab-induced cardiotoxicity, but more evidence is needed before their application in the routine practice.

## 8. Conclusion

HER2-positive breast cancer presents a heterogeneous group of diseases with various biological characteristics and clinical outcomes [66]. The management of patients with this type of breast cancer has significantly improved in recent years, and neoadjuvant treatment has become widely accepted. The introduction of trastuzumab has brought important progress in the treatment of HER2-positive breast cancer.

The neoadjuvant approach offers the possibility to reach operability in an initially inoperable disease and, in some cases, breast conservation instead of mastectomy. It also has prognostic value as patients who achieve pCR have favorable long-term outcome. The response to neoadjuvant treatment informs us of the efficacy of the used therapeutic regimen and, therefore, helps us to choose an appropriate treatment strategy. Neoadjuvant treatment requires a multidisciplinary approach, and close cooperation between surgeon, medical oncologist, radiation oncologist, and pathologist is needed.

Nowadays, sequential anthracycline-taxane-based chemotherapy in combination with trastuzumab is considered the preferred therapy for HER2-positive breast cancer in a neoadjuvant setting. However, based on the results of these trials, the dual blockage of HER2 receptor has revealed significant efficacy and presents a new therapeutic approach. Other anti-HER2 agents are under intensive investigation.

HER2 can stimulate angiogenesis through vascular endothelial growth factor upregulation. Therefore, HER2 blockage, together with inhibition of angiogenesis, could be another treatment option. In phase II study, neoadjuvant therapy with nab-paclitaxel, carboplatin and bevacizumab led to a pCR rate comparable to that found in chemotherapy/trastuzumab combinations [67].

There are many questions to be answered, for example, what is the best combination of anti-HER2 agents and with which cytostatic agents; can dual-blockage of HER2 replace the chemotherapy in some cases; and what are the reliable biomarkers for anti-HER2 therapy? Much is still to be done and other large studies are needed.
